# Circulating ex-Trm CD8 + T cells with skin-homing/chemoattraction phenotype are associated with disease severity in atopic dermatitis

**DOI:** 10.3389/fmed.2026.1656289

**Published:** 2026-02-09

**Authors:** Maria F. Ordóñez-Rubiano, Miguel Parra, Diana Bautista, Consuelo Romero-Sánchez

**Affiliations:** 1 Department of Dermatology, Hospital Militar Central, Bogotá, Colombia; 2 Cellular and Molecular Immunology Group INMUBO, Universidad El Bosque, Bogotá, Colombia; 3 Risk of Fracture, CAYRE, Bogotá, Colombia; 4 Grupo de Investigación Básica y Traslacional GIBAT, Facultad de Medicina, Universidad El Bosque, Bogotá, Colombia

**Keywords:** atopic dermatitis, atopic eczema, CLA, memory T cells, memory T lymphocytes

## Abstract

**Introduction:**

Atopic dermatitis (AD) is a chronic inflammatory skin disease driven by skin-homing memory T cells. Recent evidence suggests that a subset of tissue-resident memory T (Trm) cells can exit tissues and recirculate as ex-Trm cells.

**Methods:**

Peripheral blood memory T cell subpopulations were analyzed in adults with AD, stratified by disease severity, using multiparametric flow cytometry. CD4^+^ and CD8^+^ memory subsets and the skin-homing markers CLA, CCR4, and CCR10 were evaluated.

**Results:**

Overall CD4^+^ and CD8^+^ memory T cell distributions were preserved. AD patients showed expansion of CD4^+^ central memory T cells expressing CLA, CCR4, and CCR10. Most notably, a circulating population of CD8^+^ ex-Trm cells co-expressing CLA, CCR4, and CCR10 was increased in moderate-to-severe disease and correlated positively with clinical severity. No comparable expansion was observed for CD4^+^ ex-Trm cells.

**Discussion:**

Circulating CD8^+^ ex-Trm cells with skin-homing properties may contribute to AD progression by reseeding distant skin sites and sustaining inflammation, whereas CD4^+^ ex-Trm cells may remain preferentially retained within inflamed skin. These findings identify circulating CD8^+^ ex-Trm cells as potential biomarkers of disease severity and disease dissemination.

## 1 Introduction

Atopic dermatitis (AD) is a complex disease involving genetic predisposition, barrier dysfunction, immune activation, and environmental factors (1). AD is a heterogeneous condition, with multiple inflammatory pathways (primarily type 2) variably activated depending on race or ethnicity (2–6). Therefore, T cells are central to the pathogenesis of AD, not only during the effector phase, but also in the memory phase, where memory T cells play a key role in sustaining immune responses, recurrence, and chronicity of the disease (7).

Memory T cells are broadly classified into four major subpopulations—central memory (Tcm), effector memory (Tem), effector memory re-expressing CD45RA (Temra), and tissue-resident memory T cells (Trm)—a classification based on the expression of specific surface markers. Recent findings indicates that not all Trm cells remain permanently confined to peripheral tissues; some may exit their resident sites and re-enter the bloodstream. These so-called ex-Trm cells retain epigenetic imprints acquired during their time in barrier tissues but downregulate CD69 upon circulation (8–10).

Skin-homing T memory cells in AD are characterized by the expression of the Cutaneous Lymphocyte-Associated Antigen (CLA) and contribute to multiple aspects of AD, including pruritus, the abnormal type 2 response and, colonization and overgrowth of *Staphylococcus aureus* (*S. aureus*) (7). CLA interacts with E-selectin on cutaneous endothelial cells (7). The presence of CLA + memory T cells in peripheral blood (PB) has been proposed as a potential biomarker of AD (11, 12). Additionally, keratinocytes release C-C motif chemokine ligands (CCL) 17 and 27, whose receptors, C-C motif chemokine receptors (CCR) 4 and 10, respectively, are potential therapeutic targets and mediate T-cell skin homing/chemoattraction in the disease (12–14).

While CLA expression has been previously studied in memory T cells, the combined expression of multiple skin-homing and chemoattraction markers across different memory subsets remains poorly investigated and rarely correlated with disease severity (7, 11). Additionally, current evidence on ex-Trm cells remains limited, with few studies addressing their role in human disease (15). This gap is even more pronounced in dermatologic conditions, where studies on ex-Trm cells expressing skin-homing markers are scarce, and to date, virtually unexplored in the context of AD. These cells could potentially contribute to disease progression by reseeding distant skin sites and sustaining inflammatory activity.

Here, we characterize circulating memory T cell subpopulations in adult patients with AD, stratified by disease severity, with a special focus on expression of CLA, CCR10, and CCR4 (skin-homing markers) within these subsets, as well as on the potential existence of circulating ex-Trm cells and their association with disease severity.

## 2 Materials and methods

### 2.1 Patients and samples

An analytical cross-sectional study was conducted to characterize memory T cell subpopulations in PB of adult patients with AD, stratified by disease severity and the expression of previously described skin-homing/chemoattraction surface markers. Participants (aged 18 to 65 years) were excluded if pregnant or breastfeeding; past or current history of cancer; other autoinflammatory/autoimmune diseases; immunodeficiencies; chronic pancreatitis/liver disease; antibiotic use within the previous 3 months; and any infectious disease within the past month. Patients were treatment-free prior to sample collection: no phototherapy or systemic immunosuppressive therapies (including corticosteroids, azathioprine, methotrexate, cyclosporine, or Janus kinase inhibitors) for at least 4 weeks, and no biological therapies for at least 12 weeks. Although this was not a clinical trial, the core outcome set stablished by the Harmonizing Outcome Measure for Eczema was used to assess patients’ clinical status. AD severity was defined using the Eczema Area and Severity Index (EASI), categorized as mild (< 7), moderate (7–21), or severe (> 21) (16, 17). Patient-reported outcomes were evaluated using the Patient-oriented Eczema Measure (POEM), the peak pruritus Numerical Rating Scale over the past 24 h (pp-NRS) and the Dermatology Life Quality Index (DLQI) (17). Other scores used were SCORing Atopic Dermatitis index (SCORAD), Investigator’s Global Assessment (IGA), Mean pruritus NRS and Visual Analog Scale (VAS) for sleep disturbance. Healthy controls had no personal or family history of AD or other Th2-related conditions (asthma, allergic rhinitis, eosinophilic esophagitis, allergic conjunctivitis, or urticaria), and no history of systemic, inflammatory or infectious skin diseases. This study was conducted in accordance with the Declaration of Helsinki. Ethical approval was obtained from the institutional ethics committee (HMC 2023040, minute number: 9, date: 05-19-2023) prior to participant enrollment. Written informed consent was obtained from all participants before sample collection and clinical data acquisition.

Peripheral blood mononuclear cells (PBMCs) were isolated from 10 mL of sodium-heparinized PB samples using density gradient centrifugation with Histopaque^®^-1077, Sigma. All samples were processed within two hours of collection.

### 2.2 Flow cytometry

PBMCs were stained for viability using ViaKrome 638 (^®^-Beckman Coulter) and labeled with an 11-color panel of fluorochrome-conjugated monoclonal antibodies targeting CD3, CD4, CD8, CD45RA, CD62L, CD69, CD103, CLA, CCR4, and CCR10 (Supplementary Table 1). A median of 1 × 10^6^ events per sample was acquired for analysis.

**FIGURE 1:**
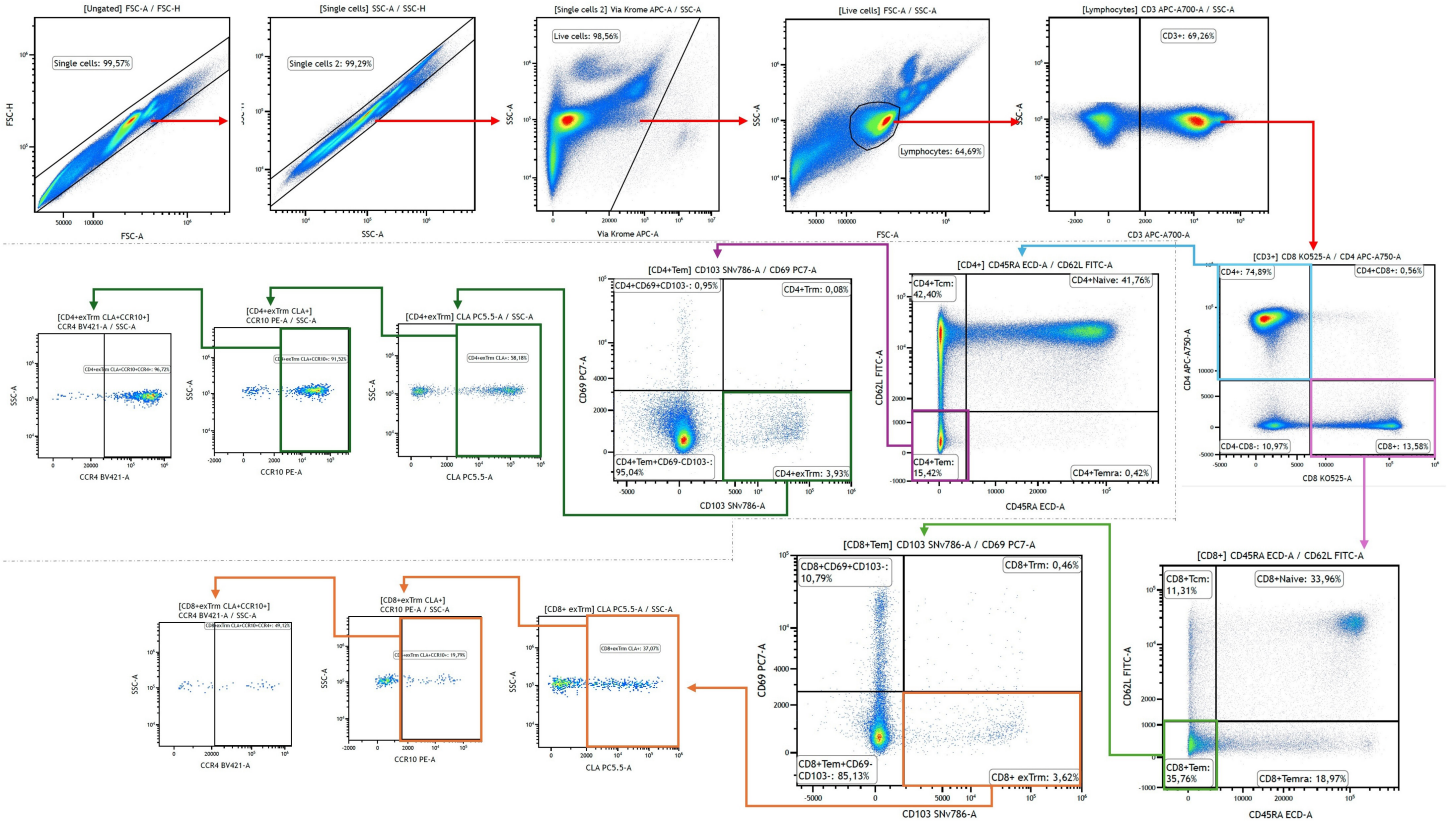
Memory T cell subpopulations were analyzed using multiparametric 11-color flow cytometry. CD3, CD4, and CD8 were used to identify total T cells and to distinguish CD4 + and CD8 + subsets. Within these, memory subsets were classified based on CD45RA and CD62L expression as follows: naïve (CD45RA + CD62L +), Tcm (CD45RA–CD62L +), Tem (CD45RA–CD62L–), and Temra (CD45RA + CD62L–). In the Tem subpopulation, expression of CD69 and CD103 was used to identify Trm (CD69 + CD103 +) and ex-Trm (CD69–CD103 +). In each memory subset, the expression of the skin-homing markers CLA, CCR10, and CCR4 was evaluated.

Data were acquired on a DxFLEX flow cytometer (Beckman Coulter, Brea, CA, United States) equipped with 3 lasers (405 nm, 488 nm, and 638 nm) using the standard configuration. Compensation was performed using single-stained PBMC samples, and critical plots were manually reviewed. List-mode data files (.fcs) were analyzed using Kaluza™ Analysis Software, version 2.3.1 (^®^-Beckman Coulter). Percentages, mean fluorescence intensity (MFI) and integrated mean fluorescence intensity (iMFI: percentage of cells positive for a marker x MFI) of each subpopulation were identified. The gating strategy is shown in Figure 1.

### 2.3 Statistical analysis

Categorical variables were summarized as frequencies and percentages, and continuous variables using central tendency and dispersion measures according to their distribution (assessed by Shapiro–Wilk test). Given the non-normal distribution of most variables, non-parametric analyses were applied. Group comparisons were performed using the Kruskal–Wallis test with Dunn’s *post hoc* correction. Associations with clinical severity scores were evaluated using Spearman’s rank correlation. For dichotomous variables, group differences were assessed using the Wilcoxon rank-sum test. Statistical significance was set at *p* < 0.05. Analyses were conducted using R version 4.3.2

## 3 Results

A total of 26 participants were included: 18 AD patients, stratified by severity into mild (*n* = 6), moderate (*n* = 7), and severe (*n* = 5) groups, and 8 healthy controls. Median age was 27 years (IQR: 10.8), with 50% of participants being female. No significant sociodemographic differences were observed between patients and controls, including marital status, education level, or socioeconomic status. Among AD patients, 77.8% reported a personal history of allergic rhinitis, 55.6% of asthma, 44.4% of allergic conjunctivitis and 16.7% of food allergies. No aeroallergen allergies were reported, and one patient had drug allergy history. There were no statistically significant differences in the prevalence of atopic comorbidities across severity subgroups (Table 1).

**TABLE 1:** Sociodemographic characteristics and comorbidities.

Variable*	Total AD (*n* = 18)	Mild (*n* = 6)	Moderate (*n* = 7)	Severe (*n* = 5)	Controls (*n* = 8)	*p*-value^Ψ^
Female sex	9 (50)	4 (66.7)	2 (28.6)	3 (60)	4 (50)	0.56
Age, median (IQR)	27 (10.8)	33 (24.2)	27 (4)	20 (26)	27 (10.75)	0.90
Cesarean delivery	3 (16.7)	2 (33.3)	1 (14.3)	0 (0)	4 (50)	0.59
Single marital status	14 (77.8)	4 (66.7)	7 (100)	3 (60)	7 (87.5)	0.27
Secondary or higher education	13 (72.22)	4 (66.7)	5 (71.4)	4 (80)	6 (75)	0.57
Middle socioeconomic status	11 (61.1)	6 (100)	3 (42.9)	2 (40)	6 (75)	0.18
**Comorbidities**						
Asthma	9 (50)	3 (50)	4 (57.1)	2 (40)	0 (0)	0.85
Allergic rhinitis	14 (77.8)	5 (83.3)	5 (71.4)	4 (80)	0 (0)	0.87
Allergic conjunctivitis	8 (44.4)	4 (66.7)	1 (14.3)	3 (60)	0 (0)	0.13
Food allergy	3 (16.7)	0 (0)	2 (28.6)	1 (20)	0 (0)	0.26
Aeroallergen allergy	0 (0)	0 (0)	0 (0)	0 (0)	0 (0)	1
Drug allergy	1 (5.56)	0 (0)	1 (14.3)	0 (0)	0 (0)	0.44

*Data are presented as n (%) unless otherwise specified.

Ψ Chi-square, Fisher’s exact test, or ANOVA applied according to data distribution. AD, atopic dermatitis; ns, not significant.

Median AD onset age was 2 years (IQR: 6.8), with 55.6% of patients reporting disease onset before 5 years of age. Median disease duration was 23 years (IQR: 19.8), with no differences observed between severity groups. Exacerbations were more common in severe AD (80%) compared to mild and moderate (*p* = 0.01). Regarding clinical phenotypes, 55.6% of patients had classic flexural, and 27.8% exhibited the head and neck variant. Clinician-Oriented Measures increased significantly with disease severity. Median EASI scores were 1.85, 11.00, and 22.10 in mild, moderate, and severe patients, respectively (*p* < 0.001). Similar trends were observed for SCORAD (*p* < 0.001) and IGA (*p* < 0.001).

Patient-reported outcomes also reflected greater disease burden in severe cases. DLQI and sleep disturbance scores were significantly higher in severe AD compared to mild and moderate cases (*p* < 0.05). Regarding treatment history, 44.4% of patients had received systemic corticosteroids and 11.1% had used cyclosporine. Topical corticosteroids were in use by 94.4% of patients at the time of sampling. Tacrolimus use was more frequent in mild AD (66.7%) than in moderate (14.3%) or severe disease (0%), reaching statistical significance (*p* < 0.05) (Table 2).

**TABLE 2:** Clinical characteristics and history of the disease.

Variable^ϕ^	Total AD (*n* = 18)	Mild (*n* = 6)	Moderate (*n* = 7)	Severe (*n* = 5)	*p*-value⟐
Age at AD onset, median (IQR)	2 (6.75)	2 (5.25)	1 (10.4)	5 (4)	0.83
Onset before 5 years of age	10 (55.6)	4 (66.7)	4 (57.1)	2 (40)	0.69
Disease duration, median (IQR)	23 (19.8)	31.5 (30.8)	21 (7)	24 (8)	0.80
Flare	5 (27.8)	0 (0)	1 (14.3)	4 (80)	0.01
Classic phenotype	10 (55.6)	2 (33.3)	5 (71.4)	3 (60)	0.63
Head and neck phenotype	5 (27.8)	4 (66.7)	1 (14.3)	0 (0)	< 0.01
**SCORES**					
EASI, median (IQR)	10.8 (17.2)	1.85 (2)	11 (4.25)	22.1 (8.8)	< 0.001
DLQI, median (IQR)	17.5 (9.5)	12.5 (6.25)	15 (9.5)	22 (9)	0.04
POEM, median (IQR)	21 (4.75)	19.5 (9.75)	21 (4)	22 (2)	0.23
ppNRS, median (IQR)	9 (1.75)	8.5 (4)	9 (2.5)	9 (1.75)	0.39
SCORAD, median (IQR)	38.7 (27.1)	22.2 (8.39)	40.1 (9.78)	61.8 (0.54)	< 0.001
IGA, median (IQR)	3 (1.75)	1.5 (1)	3 (0)	4 (0)	< 0.001
Mean pruritus NRS, median (IQR)	7.5 (2.75)	6.5 (3.25)	8 (3)	8 (2)	0.43
Sleep disturbance VAS median (IQR)	7 (5.12)	3.5 (2.5)	7 (3)	9 (4.5)	0.04
**Previous systemic treatments**					
Systemic corticosteroids	8 (44.4)	3 (50)	2 (28.6)	3 (60)	0.55
Cyclosporine	2 (11.1)	0 (0)	1 (14.3)	1 (20)	0.56
**Current topical treatments**					
Topical corticosteroids	17 (94.4)	6 (100)	7 (100)	4 (80)	0.27
Tacrolimus	5 (27.8)	4 (66.7)	1 (14.3)	0 (0)	0.03

^ϕ^Data are presented as n (%) unless otherwise specified.

⟐Kruskal-Wallis test was used. AD, atopic dermatitis; DLQI, Dermatology Life Quality Index; EASI, Eczema Area and Severity Index; IGA, Investigator’s Global Assessment; POEM, Patient-Oriented Eczema Measure; pp-NRS, peak pruritus Numeric Rating Scale; SCORAD, SCORing Atopic Dermatitis index; VAS, Visual Analog Scale; ns, not significant.

### 3.1 Total CD4^+^ and CD8^+^ T cell distribution remains unaltered in AD, but CLA^+^CCR4^+^CCR10^+^ CD8^+^ T cells are upregulated

**FIGURE 2:**
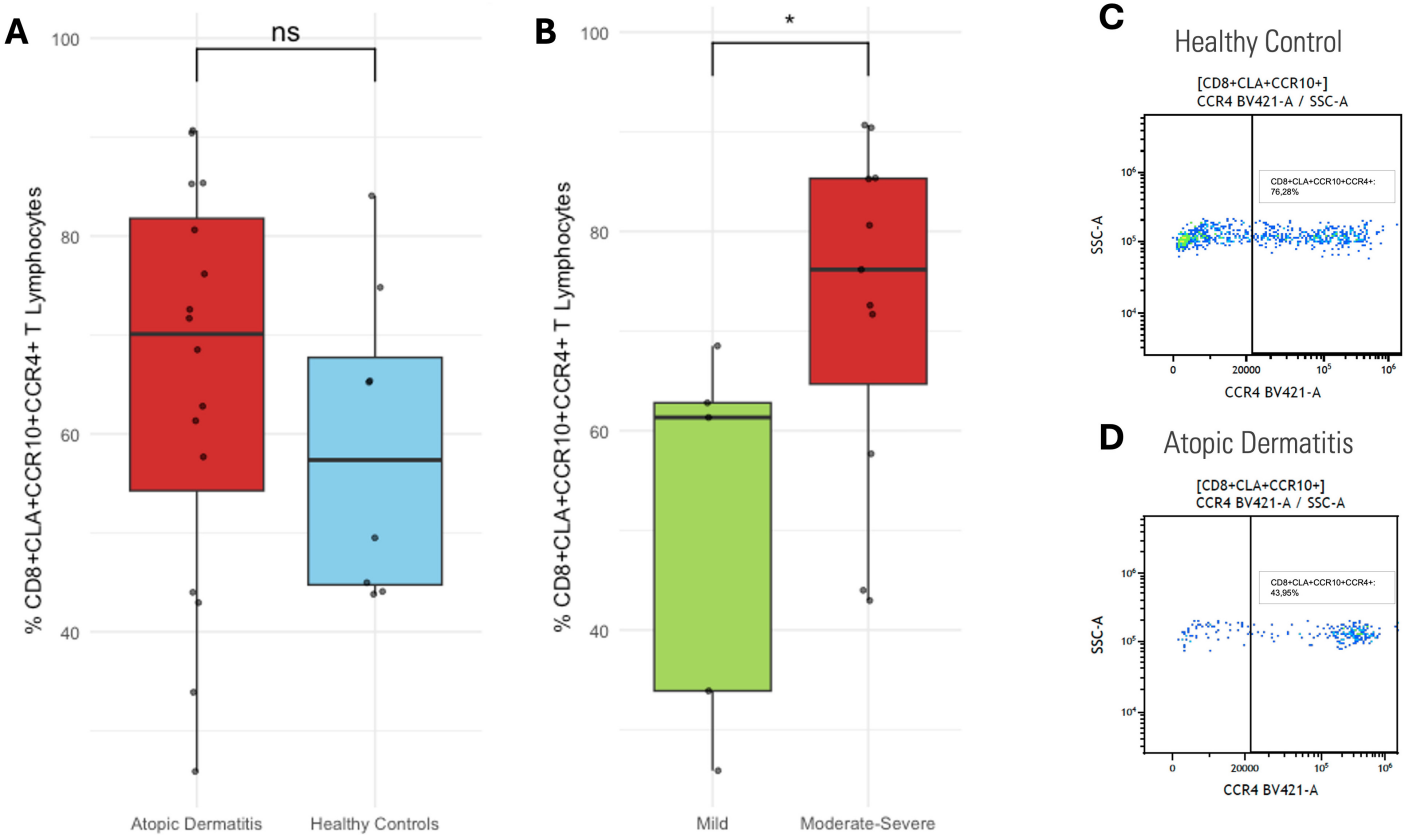
CLA^+^CCR10^+^CCR4^+^ CD8^+^ T cells. **(A)** Proportion in healthy controls vs. AD patients. **(B)** Mild vs. moderate-to-severe AD. **(C)** Representative dot plot in healthy control; and **(D)** AD patient. Percentages represent the proportion of each subset within its respective gated parent population. *p < 0.05.

Total lymphocytes and CD3^+^ T cells percentages were similar between patients with AD and healthy controls. Similarly, CD4^+^ and CD8^+^ T cells proportions showed no differences between groups, nor among AD severity subgroups. CLA expression in total CD8^+^ T cells did not differ between controls and patients, or by disease severity (Supplementary Figure 1). However, CLA^+^CCR10^+^CCR4^+^ CD8^+^ T cells percentage tended to be higher in patients, with significant increase in moderate-to-severe cases compared to mild AD (*p* < 0.05) (Figures 2A–D). In CD4^+^ T cells, CLA expression also showed no differences across groups. Although a similar distribution was observed in the CLA^+^CCR10^+^CCR4^+^ CD4^+^ subset, the differences did not reach statistical significance between patients and controls or among severity subgroups (Supplementary Figure 2).

### 3.2 Among CD4^+^ memory T cell subsets, Tcm cells show pronounced alterations in skin-homing marker expression in AD

Naïve, Tcm and Tem CD4 + T cells proportions showed no differences between patients and controls or among AD severity subgroups. In contrast, Temra CD4 + T cells (*p* < 0.05) and Trm CD4 + T cells (*p* < 0.01) percentages were significantly higher in patients compared to controls, although no differences were observed among severity groups (Supplementary Figure 3).

**FIGURE 3:**
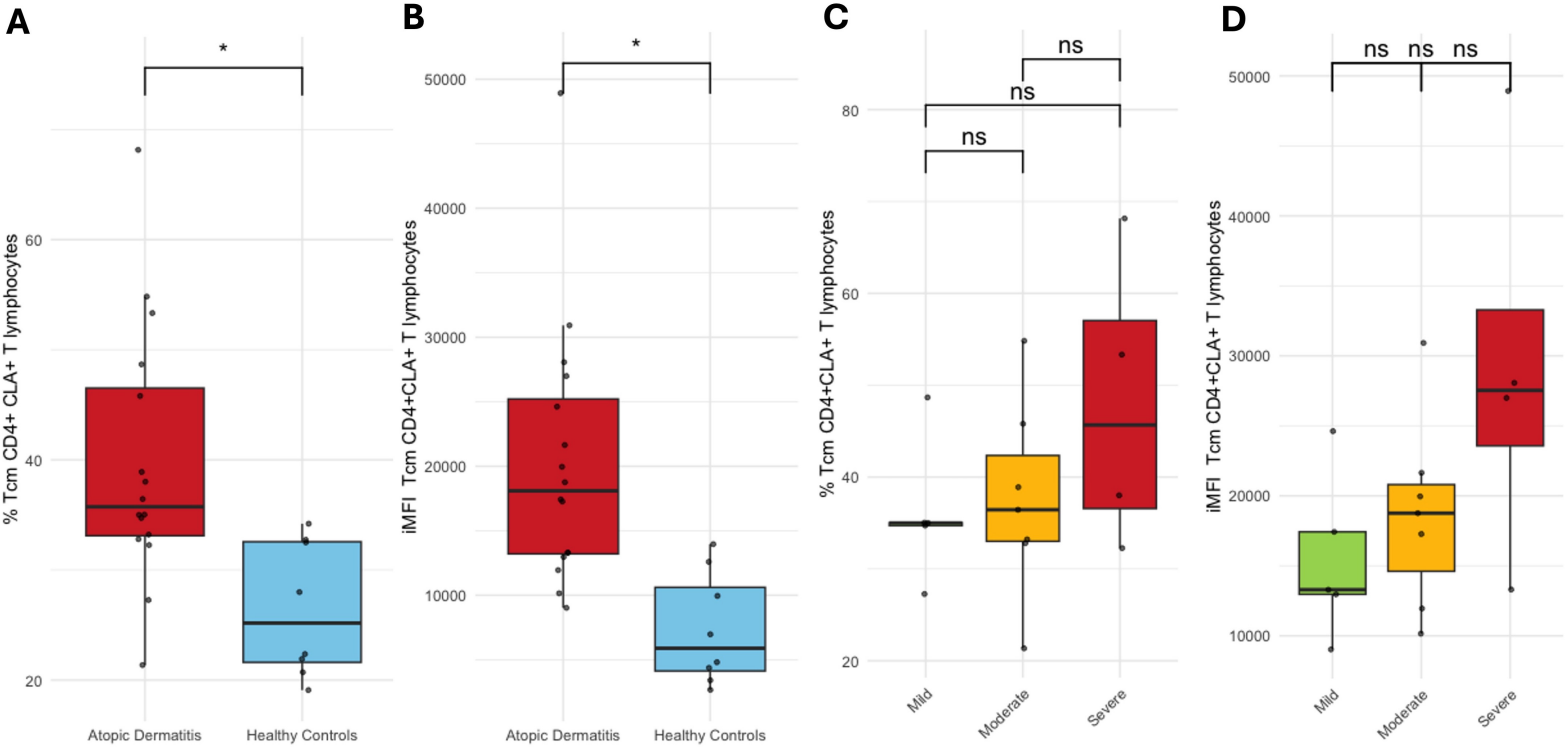
Tcm CLA^+^CD4^+^ T cells. **(A)** Proportion and **(B)** iMFI in healthy controls vs. AD patients. **(C)** Proportion and **(D)** iMFI by AD severity. Percentages represent the proportion of each subset within its respective gated parent population. *p < 0.05.

No differences in CLA expression were observed between patients and controls, nor across AD severity subgroups, in naïve and Trm CD4^+^ T cell populations (Supplementary Figure 4). In contrast, CLA^+^ Tcm CD4^+^ T cells proportion was significantly higher in patients (*p* < 0.01), and also showed higher iMFI values (*p* < 0.001). This pattern remained consistent across AD severity subgroups, but the differences did not reach statistical significance (Figures 3A–D). CLA^+^ Tem CD4^+^ T cells (*p* < 0.001) and CLA^+^ Temra CD4^+^ T cells (*p* < 0.05) percentages were lower in patients compared to controls, without variation by severity (Supplementary Figure 4).

No differences were observed in CLA^+^CCR10^+^CCR4^+^ cells proportion within naïve, Tem, Temra, or Trm CD4^+^ T cell subsets between patients and healthy controls, nor across AD severity groups. In contrast, CLA^+^CCR10^+^CCR4^+^ cells percentage was significantly higher in Tcm CD4^+^ T cells from patients (*p* < 0.01), although this difference was not maintained when stratified by disease severity (Supplementary Figure 5).

### 3.3 CLA^+^CCR10^+^CCR4^+^ frequency and expression are reduced in ex-Trm CD4^+^ T Cells in AD

**FIGURE 4:**
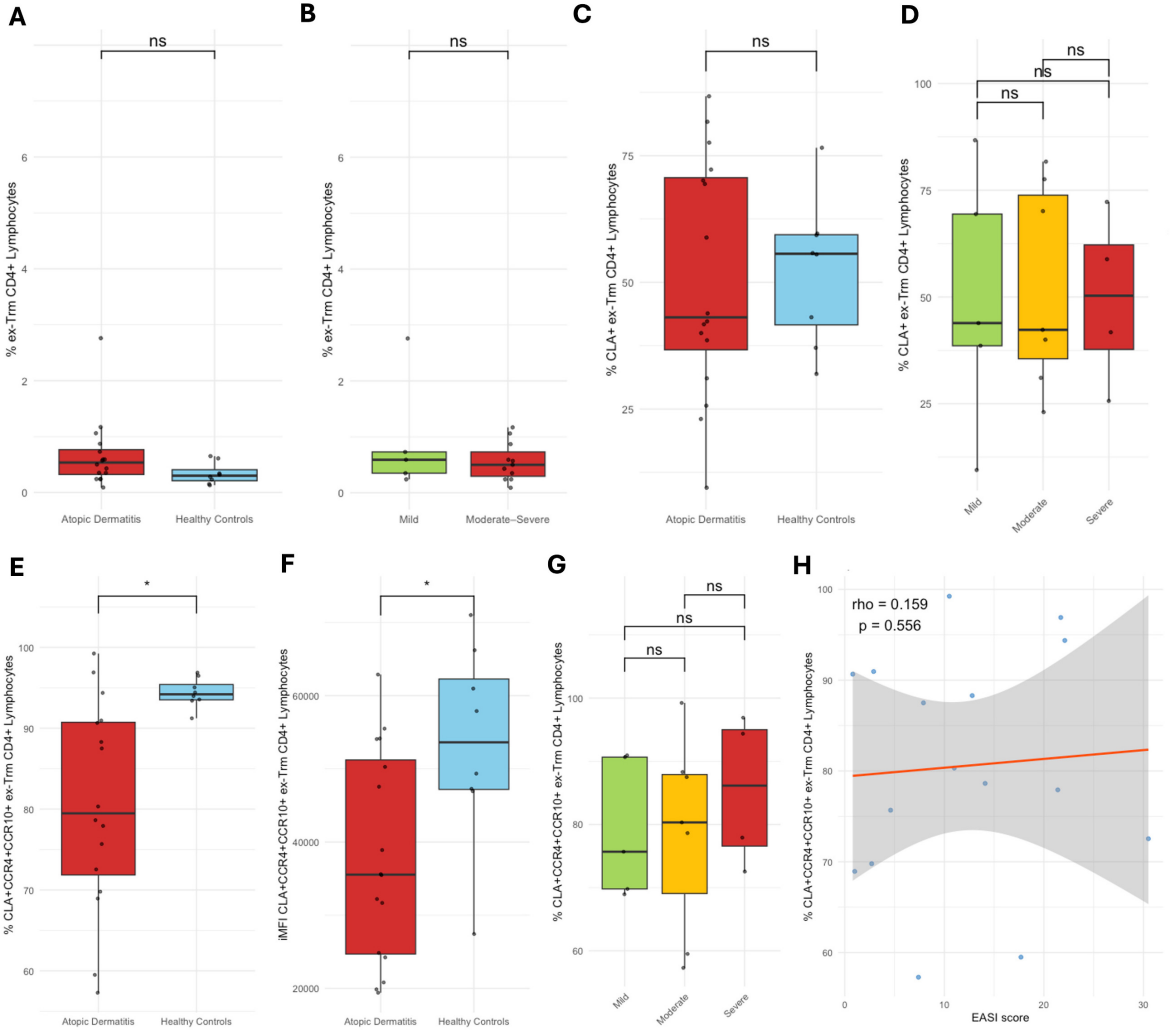
ex-Trm CD4^+^ T cells and skin-homing marker expression. **(A)** Proportion of total ex-Trm CD4^+^ T cells in healthy controls vs. AD patients; **(B)** by AD severity. **(C)** Proportion of CLA^+^ ex-Trm CD4^+^ T cells in controls vs. patients; **(D)** by AD severity. **(E)** Proportion of CLA^+^CCR10^+^CCR4^+^ ex-Trm CD4^+^ T cells in controls vs. patients. **(F)** iMFI of CLA^+^CCR10^+^CCR4^+^ ex-Trm CD4^+^ T cells in controls vs. patients. **(G)** Proportion of CLA^+^CCR10^+^CCR4^+^ ex-Trm CD4^+^ T cells by AD severity. **(H)** Spearman correlation between CLA^+^CCR10^+^CCR4^+^ ex-Trm CD4^+^ T cells and EASI score. Percentages represent the proportion of each subset within its respective gated parent population. *p < 0.05.

Ex-Trm CD4^+^ cells percentage showed no differences between patients and controls or among AD severity subgroups. Similarly, CLA^+^ ex-Trm CD4^+^ T cells proportion did not differ between groups. However, patients showed a significantly lower percentage of CLA^+^CCR10^+^CCR4^+^ ex-Trm CD4^+^ T cells compared to controls (*p* < 0.01), a finding that was also supported by iMFI analysis, which revealed reduced expression intensity in patients (*p* < 0.05). These changes were not associated or correlated with disease severity (Figures 4A–H). The frequency of ex-Trm CD4^+^CLA^+^CCR10^+^CCR4^+^ cells out of the total CD4 + T cells was 0.08, 0.08, and 0.05% in patients with mild, moderate, and severe disease, respectively, and 0.08% in healthy controls.

### 3.4 Minimal shifts in proportion and skin-homing profile of CD8^+^ Memory T cells subsets in AD

**FIGURE 5:**
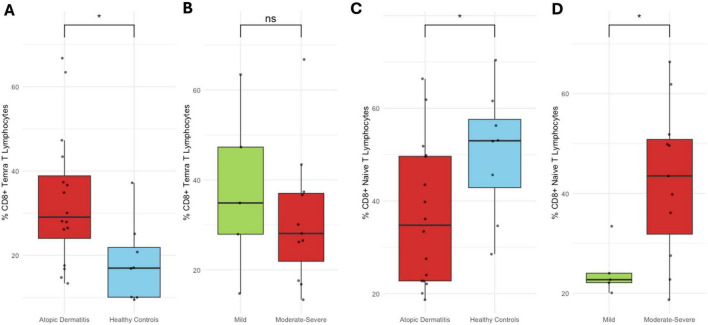
CD8^+^ Temra and naïve T cell subsets. **(A)** Proportion of CD8^+^ Temra cells in healthy controls vs. AD patients; **(B)** by AD severity. **(C)** Proportion of CD8^+^ naïve T cells in controls vs. patients; **(D)** by AD severity. Percentages represent the proportion of each subset within its respective gated parent population. *p < 0.05.

No significant differences were observed in Tcm, Tem, or Trm CD8^+^ T cells proportions between patients and healthy controls, nor across AD severity subgroups. CD8^+^ Temra cells percentage was significantly higher in patients than in controls (*p* < 0.05), although no variation was seen with disease severity (Supplementary Figure 6). Naïve CD8^+^ T cells proportion was lower in patients compared to controls (*p* < 0.05), but it increased significantly in moderate-to-severe AD compared to mild cases (*p* < 0.05) (Figures 5A–D).

No significant differences were observed in CLA^+^ CD8^+^ T cells proportion within the naïve, Tcm, Tem, Temra, or Trm subsets between patients and healthy controls, nor across AD severity groups (Supplementary Figure 7).

Across most CD8^+^ memory subsets—including Tcm, Tem, Temra, and Trm—no significant differences were found in CLA^+^CCR10^+^CCR4^+^ cells proportion between patients and controls or among AD severity groups. While CLA + CCR10 + CCR4 + naïve CD8^+^ T cells showed comparable percentages between healthy and AD individuals, a higher proportion was observed in moderate compared to mild AD, whereas values in severe AD were not significantly different. These differences, however, were not mirrored in iMFI analysis (Supplementary Figure 8).

### 3.5 CLA^+^CCR10^+^CCR4^+^ ex-Trm CD8^+^ T cells correlate with disease severity in AD

Ex-Trm CD8^+^ T cells proportion was similar between patients and healthy controls, with no significant variation across AD severity subgroups. Likewise, no differences were observed in CLA^+^ ex-Trm CD8^+^ T cells percentage between groups or by severity (Supplementary Figure 9).

**FIGURE 6:**
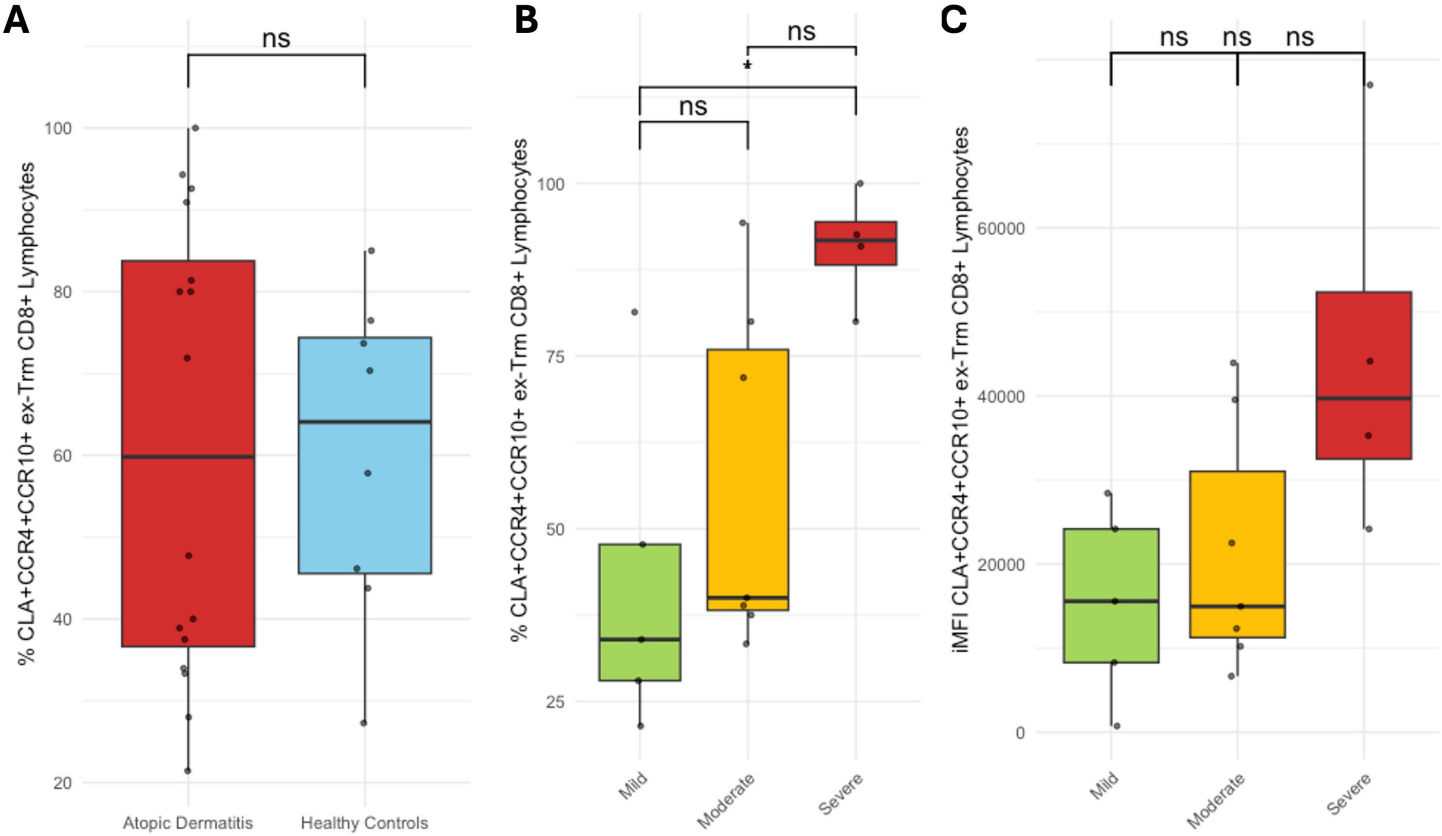
CLA^+^CCR10^+^CCR4^+^ ex-Trm CD8^+^ T cells. **(A)** Proportion in healthy controls vs. AD patients; **(B)** by AD severity. **(C)** iMFI by AD severity. Percentages represent the proportion of each subset within its respective gated parent population. *p < 0.05.

**FIGURE 7:**
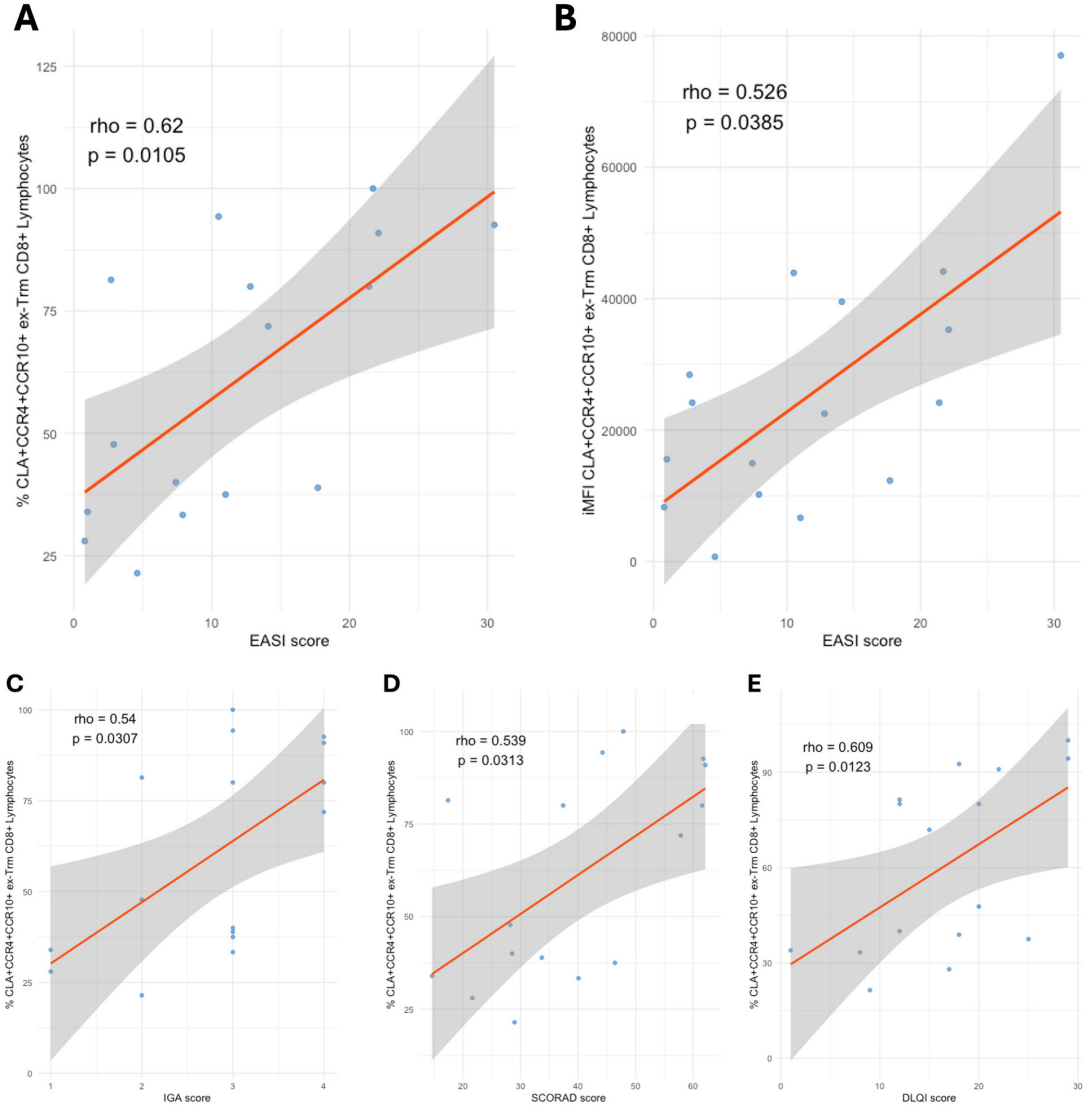
Correlations between CLA^+^CCR10^+^CCR4^+^ ex-Trm CD8^+^ T cells and clinical severity scores. **(A)** Proportion vs. EASI. **(B)** iMFI vs. EASI. **(C)** Proportion vs. IGA. **(D)** vs. SCORAD; and **(E)** vs. DLQI. All correlations were calculated using Spearman’s rank test (*p* < 0.05). Percentages represent the proportion of each subset within its respective gated parent population.

No differences were observed in CLA^+^CCR10^+^CCR4^+^ ex-Trm CD8^+^ T cells percentage between patients and healthy controls. However, when analyzed by disease severity, a progressive increase in this subset was identified, with a significantly higher proportion in severe cases compared to mild AD (*p* < 0.05), and a higher proportion in severe compared to moderate cases, although this difference did not reach statistical significance. This upward trend was also supported by iMFI analysis (Figures 6A–C). Notably, CLA^+^CCR10^+^CCR4^+^ ex-Trm CD8^+^ T cells percentage and iMFI correlated positively with disease severity as measured by the EASI score (Spearman’s rho = 0.62, *p* < 0.05; and rho = 0.526, *p* < 0.05, respectively). The percentage of this subset also showed significant positive correlations with IGA (rho = 0.54, *p* < 0.05), SCORAD (rho = 0.539, *p* < 0.05), and DLQI (rho = 0.609, *p* < 0.05) (Figures 7A–E). In contrast, no correlations or associations were found with disease duration, age at onset, or patient age; the presence of clinical flares or Th2 comorbidities; disease phenotype or socioeconomic status; nor with prior use of systemic corticosteroids or cyclosporine, or current use of topical corticosteroids (Data not shown). The frequency of ex-Trm CD8^+^CLA^+^CCR10^+^CCR4^+^ cells out of the total CD8 + T cells was 0.01, 0.02, and 0.05% in patients with mild, moderate, and severe disease, respectively, and 0.02% in healthy controls.

## 4 Discussion

Trm were thought to be a memory T cell subset that permanently reside in non-lymphoid tissue, providing localized immunosurveillance (18). Evidence demonstrating that Trm can egress from their tissue of origin, enter circulation and seed other tissues has challenged this paradigm (15). Although initially described in parabiosis murine models, human research has confirmed the phenomena, especially in ex-Trm derived from the skin, mediating Th2 and Th17 responses in hematopoietic stem cell transplantation (HSCT) (19–21). Together, ex-Trm may help explain recall responses in several previously unexplained immune phenomena, including the dissemination of skin inflammation to distant cutaneous sites (15, 22).

Parabiosis experiments in mice have shown that 15–30% of circulating memory T cells originate from ex-Trm (22, 23). Studies in humanized mice and healthy human volunteers have confirmed the presence of a small but stable population of ex-Trm in PB, accounting for approximately 1% of circulating CD4 + T cells (24). In healthy individuals, circulating CLA + T cell compartment is thought to be maintained by a steady outflow of ex-Trm from the skin (24, 25). Upon leaving the tissue, ex-Trm downregulate CD69 and are characterized by CLA and CD103 expression (24). These cells retain their tissue-specific tropism and preserve much of the transcriptional identity of their resident counterparts (21). It has been suggested that CD4^+^ Trm have a greater capacity to exit the skin and re-enter the circulation compared to CD8^+^ Trm (22). However, in a gastrointestinal infection model, antigen reencounter drives CD8^+^ ex-Trm cell expansion, with these cells differentiating into Tcm and Tem, retaining their tissue-imprinted transcriptional profile, and preserving the capacity to return to the intestine, including distant sites (26).

Our findings align with emerging evidence that skin-Trm are not permanently retained in the tissue (15). We identified an expanded population of a subset of ex-Trm CD8 + T cells expressing CLA, CCR10, and CCR4 in severe AD patients. This population showed a positive correlation with EASI, IGA, SCORAD and DLQI scores, suggesting a potential role as a circulating biomarker of disease severity.

While previous studies have focused primarily on CD4 + Trm and ex-Trm dynamics in other diseases, our findings highlight the contribution of CD8 + ex-Trm cells with a skin-homing phenotype, which may reflect active immune trafficking from inflamed skin (20, 21, 24). Their enrichment in patients with more extensive and severe disease raises the possibility that these cells contribute to eczema dissemination to distant cutaneous areas, potentially playing a role in disease progression by exiting inflamed skin, reseeding distant sites, and establishing new immune memory foci. This could explain why newly affected areas in more severe patients tend to become recurrent inflammation sites, remaining susceptible to flare-ups once involved (27). Previous studies showing that CD8^+^ Trm cells are enriched in the skin of AD patients further support the relevance of this population in sustaining local inflammatory memory. Combined with our findings, this suggests that CD8^+^ ex-Trm cells may also disseminate this memory to distant skin sites as disease severity increases (28).

In patients with ankylosing spondylitis (AS), another chronic inflammatory disease, a pathogenic role for CD8^+^ ex-Trm cells has also been described (15). In this context, a population of CD8^+^ ex-Trm cells expressing β7 integrin, CD103, and CD49a—molecules associated with gut homing—was identified and found to be increased in both PB (≈ 0.9%) and synovial fluid (≈ 7.5%) of patients with AS. These cells produced TNFα, perforin, and IL-10, suggesting a functional effector phenotype with cytotoxic and regulatory features. Based on their phenotypic similarity at both the protein and transcriptomic levels to intestinal intraepithelial lymphocytes, the authors proposed that this population originates from the gut and traffics to the joint, supporting the hypothesis of a gut–joint axis as a driver of systemic inflammation in AS (29). Together, these findings illustrate the concept that CD8^+^ ex-Trm cells may be expanded and contribute to disease progression across tissues depending on the expression of tissue-specific homing receptors.

Although classic surface markers of senescence (CD28, CD27) were not included in our multicolor panel, the design allowed us to determine that ex-Trm CD8^+^ cells were not Temra; therefore, this population is not expected to be senescent. As previously described in the context of HSCT, Trm cells can persist in the skin through the expression of senescence-associated markers; however, upon stimulation, these markers are downregulated, and the proportions of cells producing effector cytokines are comparable to those of healthy skin-derived T cells, or they may become pathogenic and contribute to graft-versus-host disease (19, 21).

CD8^+^ T cells protect mammalian hosts from intracellular infections. Persistent antigen exposure by *Herpes simplex virus* (HSV), a frequent intracellular infection in AD patients, has been shown to influence Trm biology (30, 31). Murine studies demonstrate that re-stimulated Trm can undergo retrograde migration, re-enter the circulation, and give rise to Tcm and Tem cells while retaining Trm potential (26). Other models of recurrent cutaneous viral infection have revealed pathogen-specific CD8^+^ Trm at both primary and distal skin sites, with repeated infections driving stable, skin-wide Trm populations (31, 32). These findings support HSV as a plausible factor contributing to the generation of circulating ex-Trm CD8^+^ cells with skin-homing potential in AD. Although other pathogens frequently implicated in AD, such as *Staphylococcus aureus*, *Candida*, or *Malassezia*, may also contribute to ex-Trm formation due to their chronicity, frequency, and recurrence, their predominantly extracellular biology is more likely to favor the generation of CD4^+^ rather than CD8^+^ Trm (33).

In contrast, circulating ex-Trm CD4^+^ T cells did not exhibit the same behavior. This may be explained by two mechanisms: (1) CLA^+^CCR10^+^CCR4^+^ ex-Trm CD4^+^ T cells might not be expanded, even in inflamed skin; and (2), CD4^+^ T cells actively drive local inflammation and eczematous responses in AD, as the principal effector population they may be preferentially retained in the skin. Previous studies showing that CD4^+^ Trm cells are not enriched in the skin of AD patients—even in lesional areas—reinforce the first hypothesis suggesting a distinct role in the effector phase for CD4^+^ T cells including systemic recall, rather than maintenance and expansion of local memory (28). This interpretation is further supported by our findings, as well as by others, of CLA^+^ and CLA^+^CCR10^+^CCR4^+^ CD4^+^ Tcm cells increased frequency, consistent with a systemic memory profile (11, 34–36).

We acknowledge that we identified a very low-frequency population of circulating ex-Trm cells that nevertheless reached statistical significance in relation to disease severity. Although the biological significance of these cells remains to be established, their detection by flow cytometry indicates that they can be observed in AD and that they carry skin-homing markers associated with type 2 responses. Given their rarity and the absence of functional or clonal data, these results should be viewed as an initial step, and documenting their presence provides a necessary basis for future studies to determine their physiological and potential pathogenic roles using functional assays, transcriptomics, and other state-of-the-art techniques.

This study has some limitations. The cross-sectional design precludes assessment of temporal dynamics in immune alterations. The sample size may limit the generalizability of the findings; however, it is comparable to that of other mechanistic studies in AD and was sufficient to identify consistent patterns suggesting potential differences between patients and controls, as well as across severity groups, which might have reached statistical significance in a larger cohort (5, 37, 38). Flow cytometric characterization was confined to surface markers, without evaluation of intracellular markers, cytotoxic molecules, functional assays, or clonality analyses that could help define the potential clonal status and type 1/2/3 inflammatory profile of the identified lymphocytes, as well as their cytotoxic capacity and potential contribution to tissue damage in AD. The levels of plasma chemokines were not assessed, which would provide valuable insight into the systemic effects that skin-derived chemokines may exert on the subsets of memory lymphocytes expressing skin-homing receptors that we identified.

Notable strengths of this study include stringent patient selection criteria, enabling analysis of immune parameters in untreated individuals—providing a clearer view of disease physiology without the confounding effects of recent therapeutic interventions. Additionally, all participants underwent a comprehensive and detailed clinical evaluation, allowing for precise phenotypic and immunological correlations.

Future longitudinal studies should evaluate CLA^+^CCR10^+^CCR4^+^ ex-Trm CD8^+^ T cells dynamics during disease flaring, treatment response, or overall disease course. Simultaneous assessment of both compartments—skin and PB—could offer valuable insight into pathophysiology of these poorly characterized cells, and into their behavior between affected skin and circulation, in both CD4^+^ and CD8^+^ ex-Trm populations. Furthermore, functional assays or single-cell transcriptomic analyses of these cells may elucidate pathogenic roles, whether they represent valid therapeutic targets or biomarkers of disease severity in AD.

Finally, we identified a circulating CLA^+^CCR4^+^CCR10^+^ CD8^+^ ex-Trm subset, whose enrichment in more severe patients suggests a role in disease dissemination and progression in AD, highlighting these cells—largely overlooked in skin disease models and human studies—as potential biomarkers of disease severity and therapeutic targets.

## Data Availability

The raw data supporting the conclusions of this study are available from the corresponding author upon reasonable request, in accordance with ethical and data protection requirements.
