# Supplementary material for: Circulating ex-Trm CD8 + T cells with skin-homing/chemoattraction phenotype are associated with disease severity in atopic dermatitis
Source: Front Med (Lausanne). 2026 Feb 9;13:1656289. doi: 10.3389/fmed.2026.1656289 (PMC12926171; doi:10.3389/fmed.2026.1656289)
Supplement: Supplementary file 1 [file Data_Sheet_1.pdf]

Supplementary Material

1 Supplementary Figures and Tables

Supplementary Figure 1

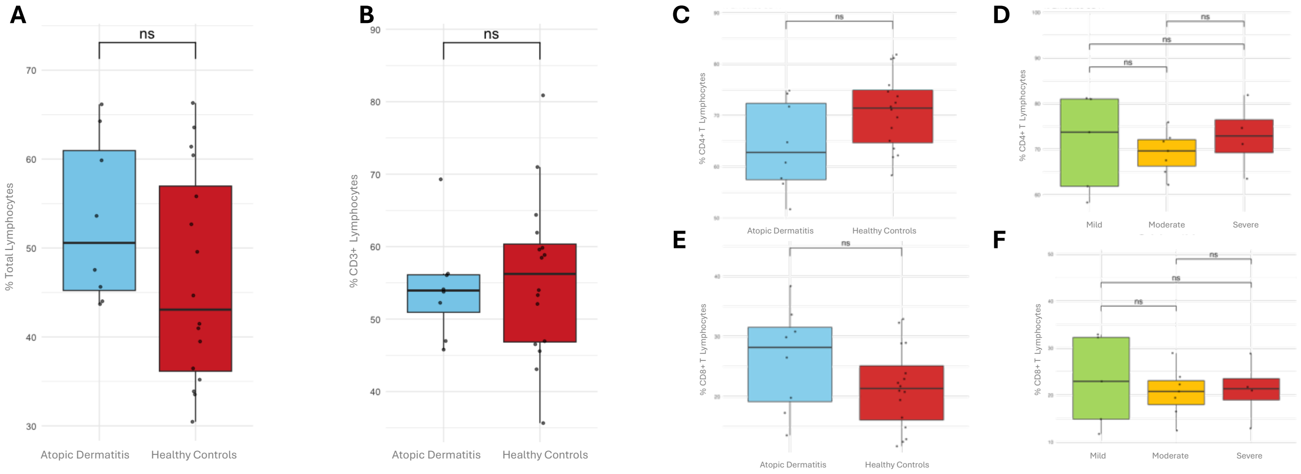

Supplementary Figure 2

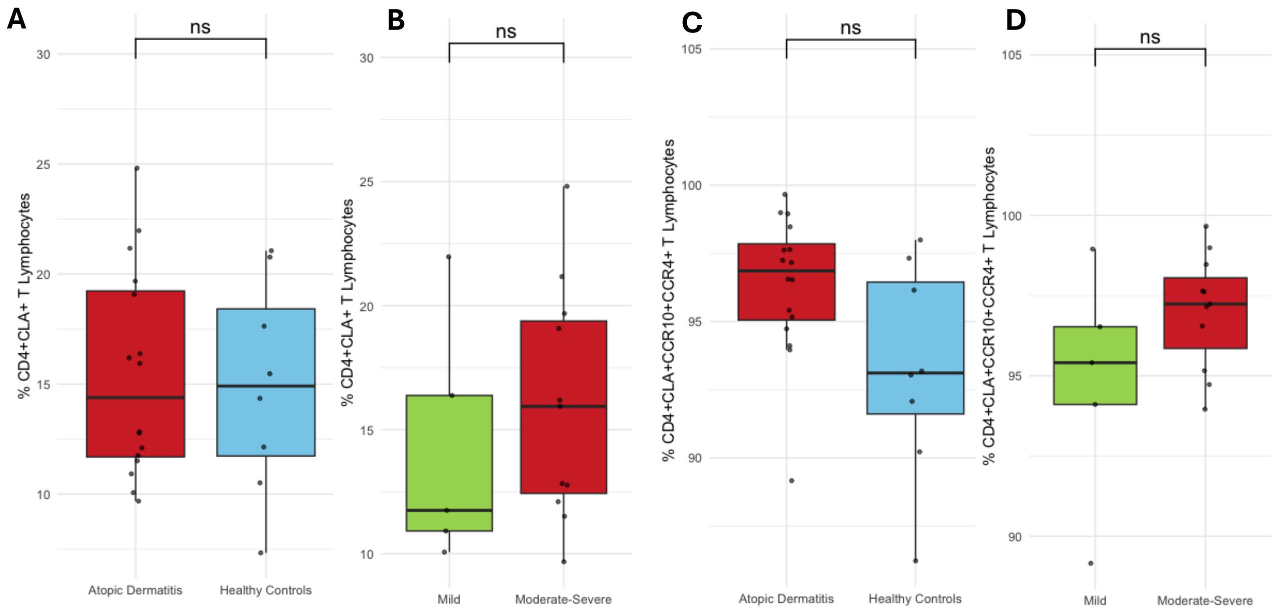

Supplementary Figure 3

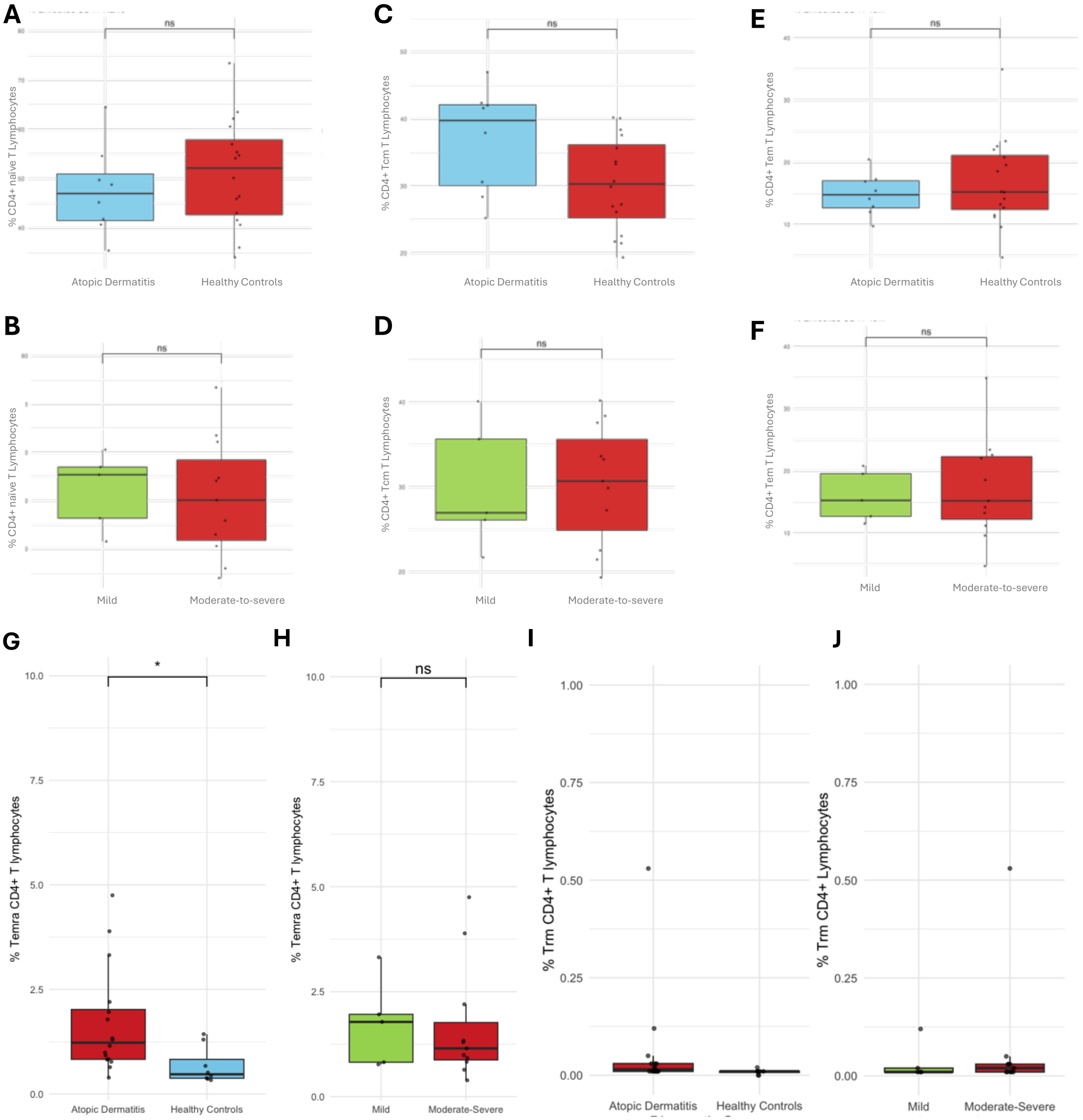

Supplementary Figure 4

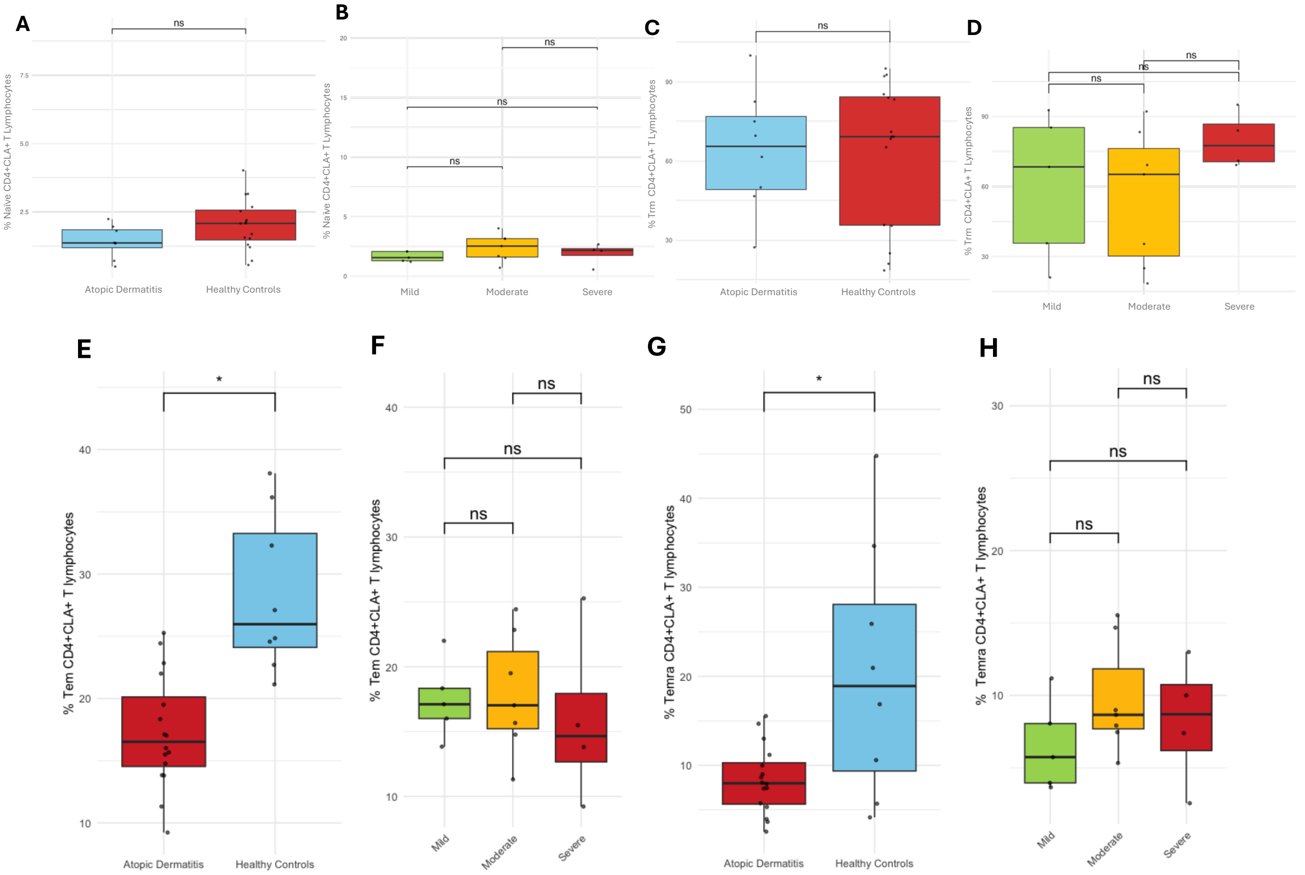

Supplementary Figure 5

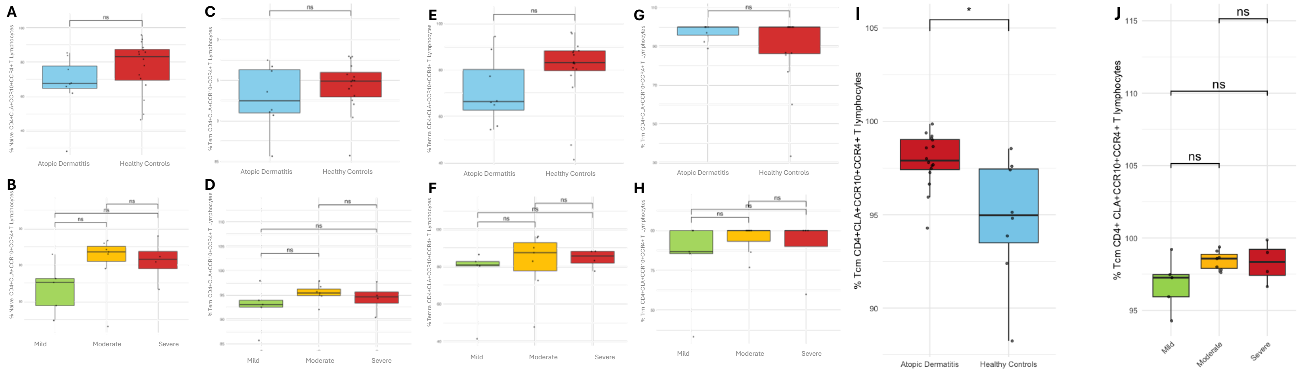

Supplementary Figure 6

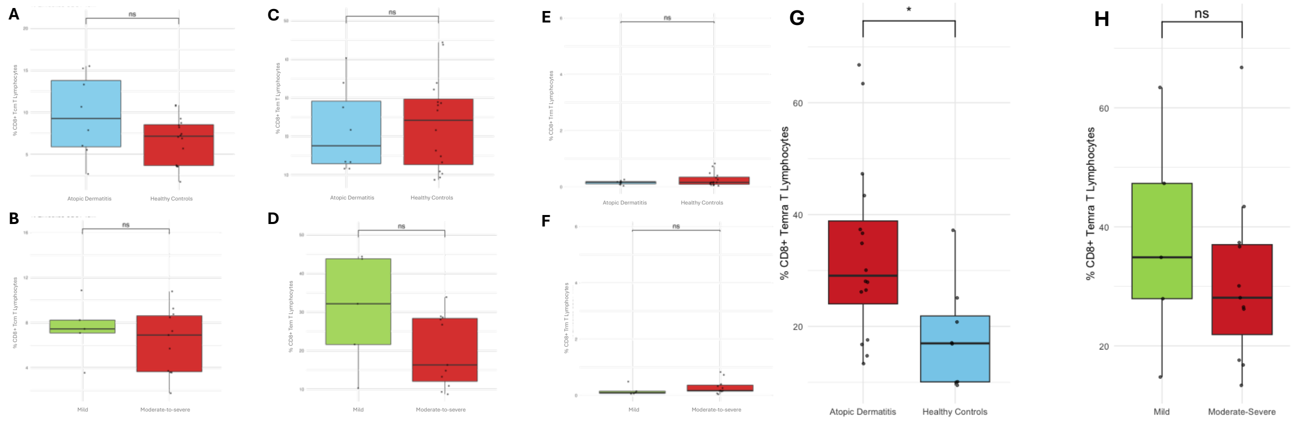

Supplementary Figure 7

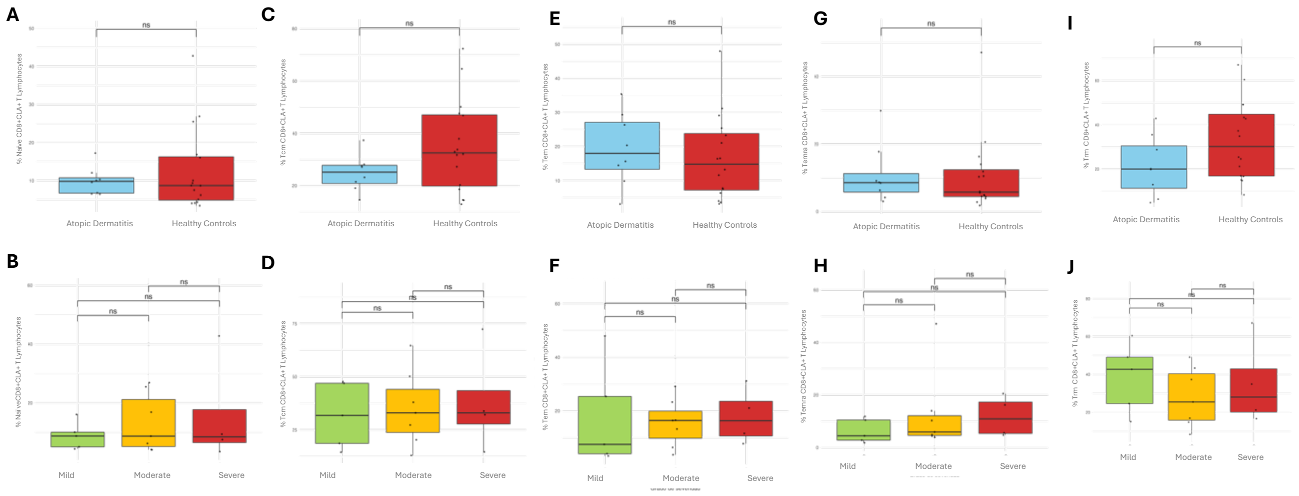

Supplementary Figure 8

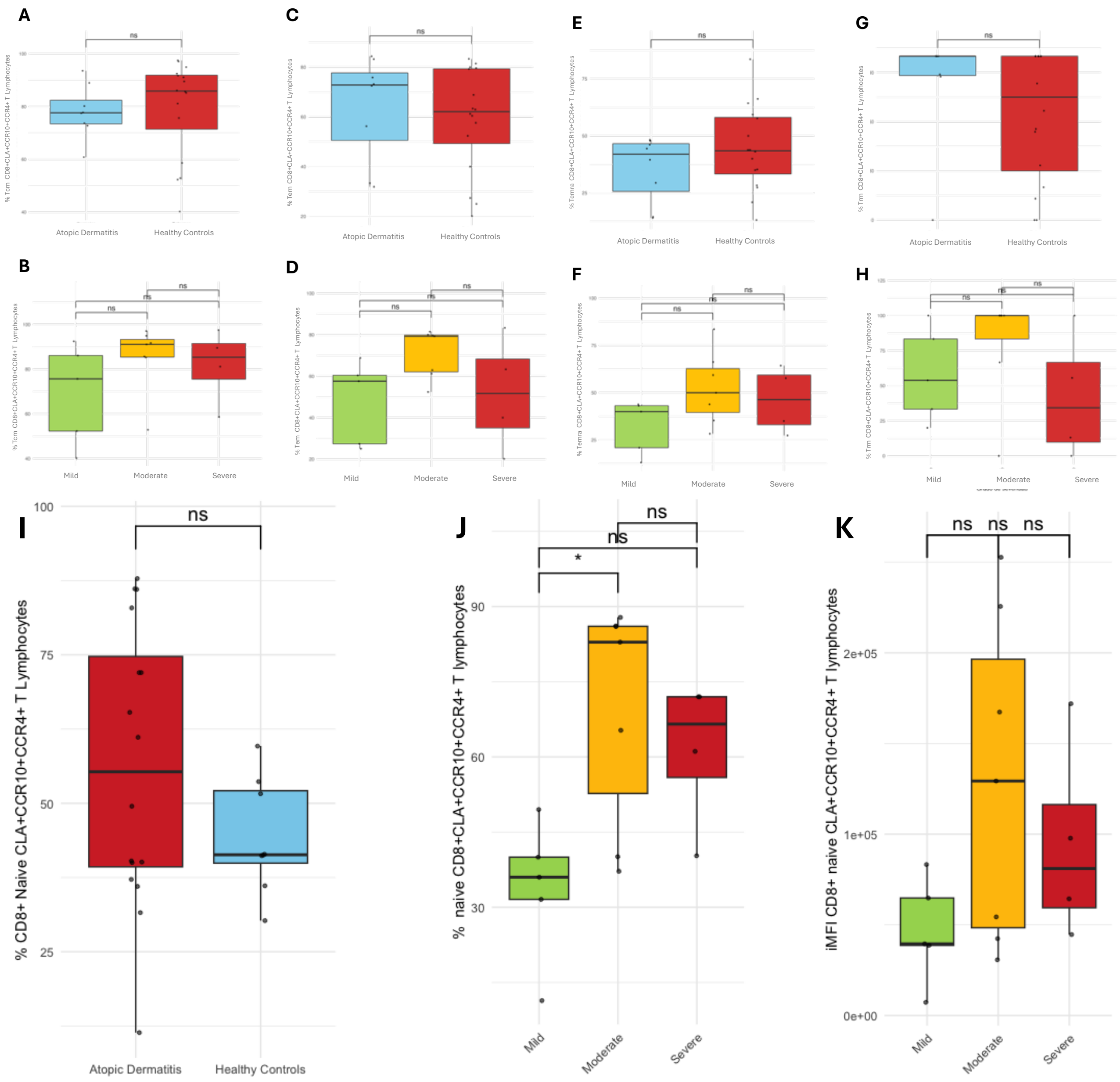

Supplementary Figure 9

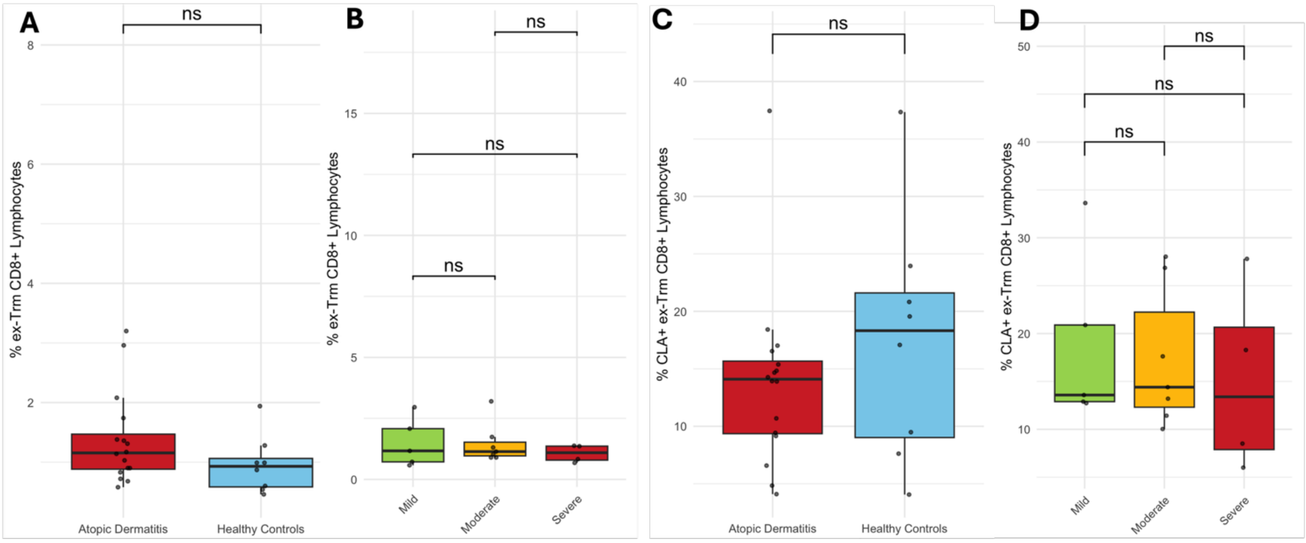

Supplementary Table 1. 11-color panel of fluorochrome-conjugated monoclonal antibodies										
Molecule	Fluorochrome	Vendor	Laser (nm)	Detector (bandpass)	Brightness	Clone	Catalog #	Host	Isotype	Target Species
CD3	A700	Beckman Coulter	638	712/25	4	UCHT1	B10823	Mouse	IgG1	Human
CD4	A750	Beckman Coulter	638	780/60	4	13B8.2	A94682	Mouse	IgG1	Human
CD8	Krome Orange	Beckman Coulter	405	525/40	4	B9.11	B00067	Mouse	IgG1	Human
CD62L	FITC	Beckman Coulter	488	525/40	2	DREG56	IM1231U	Mouse	IgG1	Human
CD45RA	ECD	Beckman Coulter	488	610/20	–	2H4	B49193	Mouse	IgG1	Human
CCR10	PE	BD Biosciences	488	585/42	5	1B5	563656	Mouse	IgG2a, κ	Human
CLA	PerCP5.5	BioLegend	488	690/50	3	HECA-452	321314	Rat	IgG1, κ	Human –Mouse
CCR4	BV421	BioLegend	405	450/45	5	L291H4	359414	Mouse	IgG1, κ	Human
CD69	PC7	Beckman Coulter	488	780/60	4	TP1.55.3	A80710	Mouse	IgG2b	Human
CD103	SNv786	Beckman Coulter	405	780/60	–	2G5	C78085	Mouse	IgG1	Human
Viability dye	APC	Beckman Coulter	638	660/10	2	–	C36624	–	–	–

1.1 Supplementary Figures and Table legends

Supplementary Figure 1. Proportion of total Lymphocytes and T cell subsets. (A) Total lymphocytes in healthy controls vs. AD patients. (B) CD3+ lymphocytes in healthy controls vs. AD patients (C) CD4+ T lymphocytes in healthy controls vs. AD patients. (D) by AD severity. (E) CD8+ T lymphocytes in healthy controls vs. AD patients. (D) by AD severity.

Supplementary Figure 2. CLA^+CD4^+ T cells. (A) Proportion in healthy controls vs. AD patients. (B) mild vs. moderate-to-severe AD. $CLA^+CCR10^+CCR4^+$ $CD4^+$ T cells. (C) Proportion in healthy controls vs. AD patients. (D) mild vs. moderate-to-severe AD. Percentages represent the proportion of each subset within its respective gated parent population.

Supplementary Figure 3. Proportion of different $CD4^+$ T cells subsets. (A) Naive in healthy controls vs. AD patients; (B) mild vs. moderate-to-severe AD**.**. (C) Tcm in healthy controls vs. AD patients; (D) mild vs. moderate-to-severe AD. (E) Temra in healthy controls vs. AD patients; (F) mild vs. moderate-to-severe AD**.**. (G) Tem in healthy controls vs. AD patients; (H) mild vs. moderate-to-severe AD**.**. (I) Trm in healthy controls vs. AD patients; (J) mild vs. moderate-to-severe AD. Percentages represent the proportion of each subset within its respective gated parent population.

Supplementary Figure 4. Proportion of CLA^+ in different $CD4^+$ T cells subsets (A) Naive cells in healthy controls vs. AD patients; (B) by AD severity (C) Trm in healthy controls vs. AD patients; (D) by AD severity. (E) Tem in healthy controls vs. AD patients; (F) by AD severity (G) Temra in healthy controls vs. AD patients; (H) by AD severity. Percentages represent the proportion of each subset within its respective gated parent population.

Supplementary Figure 5. Proportion of $CLA^+CCR10^+CCR4^+$ in different $CD4^+$ T cells subsets. (A) Naive in healthy controls vs. AD patients; (B) by AD severity. (C) Tem in healthy controls vs. AD patients; (D) by AD severity. (E) Temra in healthy controls vs. AD patients; (F) by AD severity. (G) Trm in healthy controls vs. AD patients; (H) by AD severity. (I) Tcm in healthy controls vs. AD patients; (J) by AD severity. Percentages represent the proportion of each subset within its respective gated parent population.

Supplementary Figure 6. Proportion of different $CD8^+$ T cells subsets. (A) Tcm in healthy controls vs. AD patients; (B) mild vs. moderate-to-severe AD.. (C) Tem in healthy controls vs. AD patients; (D) mild vs. moderate-to-severe AD. (E) Trm in healthy controls vs. AD patients; (F) mild vs. moderate-to-severe AD.. (G) Temra in healthy controls vs. AD patients; (H) mild vs. moderate-to-severe AD.Percentages represent the proportion of each subset within its respective gated parent population.

Supplementary Figure 7. Proportion of CLA^+ in different $CD8^+$ T cells subsets (A) Naive cells in healthy controls vs. AD patients; (B) by AD severity (C) Tcm in healthy controls vs. AD patients; (D) by AD severity. (E) Tem in healthy controls vs. AD patients; (F) by AD severity (G) Temra in healthy controls vs. AD patients; (H) by AD severity. (I) Trm in healthy controls vs. AD patients; (J) by AD severity. Percentages represent the proportion of each subset within its respective gated parent population.

Supplementary Figure 8. Proportion of $CLA^{+}CCR10^{+}CCR4^{+}$ in different $CD8^{+}$ T cells subsets. (A) Tcm in healthy controls vs. AD patients; (B) by AD severity. (C) Tem in healthy controls vs. AD patients; (D) by AD severity. (E) Temra in healthy controls vs. AD patients; (F) by AD severity. (G) Trm in healthy controls vs. AD patients; (H) by AD severity(I) Naive in healthy controls vs. AD patients; (J) by AD severity. (K) iMFI by AD severity. Percentages represent the proportion of each subset within its respective gated parent population.

Supplementary Figure 9. (A) Proportion of total ex-Trm $CD8^{+}$ T cells in healthy controls vs. AD patients; (B) by AD severity (C) Proportion of ex-Trm $CD8^{+}$CLA+ T cells in healthy controls vs. AD patients; (D) by AD severity. Percentages represent the proportion of each subset within its respective gated parent population.

Supplementary Table 1. Antibody clones, fluorochromes, catalog numbers, and detector configuration
